# Understanding the gap between access and use: a qualitative study on barriers and facilitators to insecticide-treated net use in Ghana

**DOI:** 10.1186/s12936-019-3051-0

**Published:** 2019-12-12

**Authors:** Collins Stephen Ahorlu, Philip Adongo, Hannah Koenker, Sixte Zigirumugabe, Solomon Sika-Bright, Eric Koka, Philip Teg-Nefaah Tabong, Danielle Piccinini, Sylvester Segbaya, Bolanle Olapeju, April Monroe

**Affiliations:** 1 Department of Epidemiology, Noguchi Memorial Institute for Medical Research, College of Health Sciences, University of Ghana, Legon, Ghana; 2 Department of Social and Behavioral Science, School of Public Health, College of Health Sciences, University of Ghana, Legon, Ghana; 3 PMI VectorWorks Project, Johns Hopkins Center for Communication Programs, Baltimore, MD USA; 4 U.S. President’s Malaria Initiative, U.S. Agency for International Development, Accra, Ghana; 5 Department of Sociology and Anthropology, University of Cape Coast, Cape Coast, Ghana; 6 Johns Hopkins Center for Communication Programs, Accra, Ghana; 7 Swiss Tropical and Public Health Institute, Basel, Switzerland; 8 University of Basel, Basel, Switzerland

**Keywords:** Malaria, Ghana, Insecticide-treated mosquito net, Prevention

## Abstract

**Background:**

Mass and continuous distribution channels have significantly increased access to insecticide-treated nets (ITNs) in Ghana since 2000. Despite these gains, a large gap remains between ITN access and use.

**Methods:**

A qualitative research study was carried out to explore the individual and contextual factors influencing ITN use among those with access in three sites in Ghana. Eighteen focus group discussions, and free listing and ranking activities were carried out with 174 participants; seven of those participants were selected for in-depth case study. Focus group discussions and case study interviews were audio-recorded, transcribed verbatim, and analysed thematically.

**Results:**

ITN use, as described by study participants, was not binary; it varied throughout the night, across seasons, and over time. Heat was the most commonly cited barrier to consistent ITN use and contributed to low reported ITN use during the dry season. Barriers to ITN use throughout the year included skin irritation; lack of airflow in the sleeping space; and, in some cases, a lack of information on the connection between the use of ITNs and malaria prevention. Falling ill or losing a loved one to malaria was the most powerful motivator for consistent ITN use. Participants also discussed developing a habit of ITN use and the economic benefit of prevention over treatment as facilitating factors. Participants reported gender differences in ITN use, noting that men were more likely than women and children to stay outdoors late at night and more likely to sleep outdoors without an ITN.

**Conclusion:**

The study results suggest the greatest gains in ITN use among those with access could be made by promoting consistent use throughout the year among occasional and seasonal users. Opportunities for improving communication messages, such as increasing the time ITNs are aired before first use, as well as structural approaches to enhance the usability of ITNs in challenging contexts, such as promoting solutions for outdoor ITN use, were identified from this work. The information from this study can be used to inform social and behaviour change messaging and innovative approaches to closing the ITN use gap in Ghana.

## Background

Malaria remains a major public health problem in Ghana. In 2016, malaria accounted for 39% of outpatient attendance, 25% of all admissions and 4% of all deaths. Of those deaths, nearly half were among children less than 5 years old [1]. The national malaria parasite prevalence in 2016 was 20%, ranging between 5% in the Greater Accra Region to 31% in the Eastern Region [2], an indication that Ghana remains within the control phase of the malaria control-elimination continuum [3].

In Ghana, the main vectors of malaria are *Anopheles gambiae* complex and *Anopheles funestus* group [4]. These vectors are mostly anthropophilic with a preference for indoor biting and resting, with some variation among subspecies [5]. The main parasite causing malaria in Ghana is *Plasmodium falciparum*, which is responsible for > 85% of malaria cases [6]. The other malaria parasites are *Plasmodium malariae* and *Plasmodium ovale* [7].

Insecticide-treated nets (ITNs) are a major intervention of global malaria control initiatives based on overwhelming evidence showing significant reductions in malaria-related morbidity and mortality as a result of ITN use [8, 9]. These studies stimulated efforts to increase ITN ownership in many malaria-endemic countries, driven largely by donor-funded ITN distribution programmes [10–13]. Though access to ITNs has increased substantially in affected countries and rates of use have also improved, some countries continue to have below target use among those with access to ITNs, and seasonal variability in ITN use practices [14–16].

Since 2000, Ghana has made significant progress toward increasing ITN access through mass and continuous distribution channels [17, 18]. Despite these gains, ITN use remains sub-optimal in some settings. National household surveys provide useful information on the levels of use given access and the differences in these levels across groups in Ghana. A secondary analysis of the 2011 Multiple Indicator Cluster Survey, 2014 Ghana Demographic and Health Survey (DHS), and 2016 Malaria Indicator Survey (MIS) data looked at net use given access across region, sex, age, and wealth quintile. Overall, 63% of Ghanaians with access to an ITN in their household reported using one the night before the interview; however, significant variation was observed across geographic zones and rural/urban settings. In many central areas of Ghana, between 60% and 80% of those with access to an ITN reported using it, compared to 20% to 40% along the coast, with a particularly low use to access ratio in and between the urban areas of Accra and Takoradi. The analysis revealed that ITN use among children under 5 years was highest compared to the general population, while ITN use was lowest among males aged 15 to 49 years. When controlling for other factors, household supply of ITNs, wealth quintile, and residence were the most important factors associated with ITN use [19].

While national surveys provide useful quantitative estimates of ITN use trends in Ghana, and differences across groups, they do not offer insights into the key factors driving use and non-use of available ITNs. To fill this gap, a qualitative research study was carried out to provide in-depth information on barriers and facilitators to ITN use among those with access.

## Methods

### Study sites

**Fig. 1:**
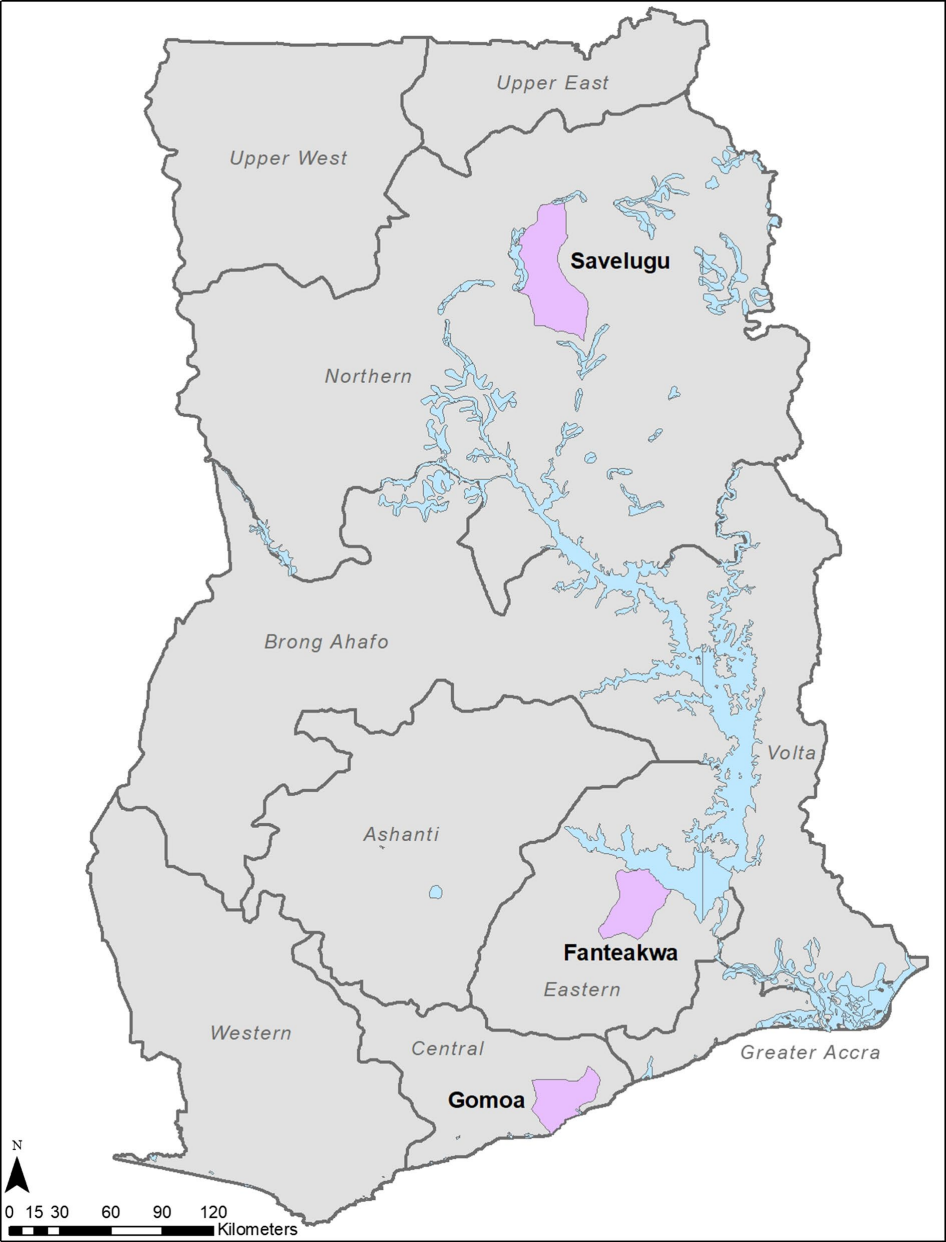
Map of Ghana Showing Study Districts

Data were collected in March 2018, concurrently across three study sites. The 10 administrative regions of Ghana were divided into zones, based on the three ecological areas (the Coastal savannah ecological zone included the Volta, Greater Accra, Central and Western Regions; the Forest ecological zone included the Eastern, Ashanti, and Brong-Ahafo Regions; the Savannah ecological zone included the Upper East, Upper West, and the Northern Regions). One region was selected from each of three ecological zones based on a low ITN use-access ratio calculated from the 2016 MIS. A district was purposively selected from each of the three regions in consultation with the Ghana National Malaria Control Programme. Gomoa West District in the Central Region (Coastal Savanna zone), Fanteakwa District in the Eastern Region (Forest zone) and Savelugu District in the Northern Region (Northern Savanna zone) were selected (Fig. 1). The selected districts were viewed as generally representative of their respective regions. In each district, an urban community, the district capital, and a rural community were selected for data collection.

### Data collection

#### Community entry

Prior to initiation of data collection, the study team informed regional, district and health facility officials of the Ghana Health Service (GHS) in the selected study sites about the study objectives, methods, and timeline. Upon arrival in the community, the study team met the GHS officials who facilitated meetings with local leaders. After gaining the support of the community leaders, the team, assisted by community-based volunteers and an officer of the district health management team, recruited study participants. Participants were purposively selected based on the following categorizations: community leaders, health workers employed at the local health clinic, caretakers of children under-five, male community members ages 18–49, female community members ages 18–49, and male and female community members ages 50 or older. To be included in the study, all participants had to own at least one ITN in their household.

#### Questionnaire

Prior to focus group discussions, all participants filled out a brief questionnaire with information on age, sex, number of ITNs in their household, number of household members, and self-reported level of ITN use. Level of use was categorized as regular user, defined as using an ITN every night throughout the year; occasional user, defined as using an ITN some nights throughout the year; seasonal user, defined as using an ITN during rainy season only; and non-user, defined as someone who never uses an ITN.

#### Focus group discussions

Focus group discussion (FGD) was the primary data collection technique and was used to explore individual and contextual factors influencing ITN use among those with access to ITNs. Free listing and ranking activities were incorporated into the FGD, where at the end of each discussion, participants were asked to list all the motivators and barriers to ITN use in their households and in the community-at-large. After the listing, they were asked to rank them individually. At the end of the individual ranking, participants were brought together to discuss their most important motivators and barriers and arrive at a group consensus.

Trained and experienced FGD moderators led the sessions in the local languages; health worker FGDs were conducted in English. A moderator, note taker, and supervisor facilitated each FGD. The moderator followed a semi-structured discussion guide, developed around key themes of interest. Each FGD lasted approximately 90 to 150 min. The discussions were audio-recorded and transcribed verbatim into English on an on-going basis. Following each day’s work, the study teams debriefed within and across sites to identify emerging themes and ensure data quality across the three sites. Supervisors reviewed all English translations and provided continuous feedback to the data collection teams.

#### Case studies

To complement data from FGDs, a subset of FGD participants were selected for in-home case studies in each zone. Cases were selected to represent a range of ITN use practices. Participants were interviewed to build on information obtained from FGD sessions. Observations were done on the general sleeping arrangements; availability, location, and physical condition of ITNs. While there is a standard methodology for measuring ITN durability, the focus of this study was to observe and describe the condition of ITNs in a small number of homes at one point in time as part of the qualitative study. This included whether ITNs were hung on sleeping places or not and whether they had visible holes in them at the time of observation. Field notes were taken using a semi-structured template and the interviews were audio-recorded. Audio recordings were transcribed verbatim on an ongoing basis.

#### Data analysis

Data were analysed using an iterative process, beginning during data collection through daily debriefings and review of data and emergent themes. NVivo 12 (QSR International), a qualitative data analysis software program for coding, storage, indexing, and retrieval, was used to thematically analyse the FGD and case study transcripts [20]. Coding was carried out using predetermined themes based on the research questions and key areas of interest [21]. The codebook was developed and refined by coauthors Dr. Eric Koka, Professor Collins Stephen Ahorlu, and Professor Philip Adongo. Coding of all transcripts was carried out by Dr. Philip Teg-Nefaah Tabong and reviewed by Professor. Ahorlu and Professor Adongo. Visualizations were generated for the most common barriers and facilitators to ITN use by inputting content from the free-listing and ranking activity into Word Art, a free, online word cloud creator.

Access to ITNs was calculated using the approach put forward by Kilian et al. and endorsed by the Roll Back Malaria Monitoring and Evaluation Reference Group [22, 23]. To calculate the potential ITN users, study team members multiplied the number of ITNs in each of the participant’s household by two. If the number of potential users was larger than the number of household members, the number of household members was used. To calculate access, potential ITN users were divided by the total number of people in study participants’ households.

#### Ethical approval

The study was reviewed by the Johns Hopkins University Institutional Review Board and met the criteria for exemption (IRB No. 8437). The study was reviewed and approved by the Noguchi Memorial Institute for Medical Research (IRB No.1276) and the Ghana Health Service Ethics Review Committee (GHS-ERC: 007/02/18). All participants provided written informed consent prior to participation.

## Results

A total of 18 focus group discussions were carried out, six in each study site, with 174 total participants. Table 1 includes the breakdown of the number of participants in each category of focus group, by region. Participants were roughly evenly split between male (43%) and female (57%) and urban (53%) and rural (47%) communities in the selected districts.

**Table 1:** Participants by focus group category and region

	Central # of groups (participants)	Eastern # of groups (participants)	Northern # of groups (participants)	Total
Health workers	1 (8)	1 (10)	1 (9)	3 (27)
Community leaders	1 (9)	1 (12)	1 (9)	3 (30)
Males ages 18 to 49	1 (10)	1 (12)	1 (10)	3 (32)
Females ages 18 to 49	1 (8)	1 (12)	1 (10)	3 (30)
Caretakers of children > 5	1 (9)	1 (8)	1 (10)	3 (27)
Adults > 49	1 (10)	1 (9)	1 (9)	3 (28)
	6 (54)	6 (63)	6 (57)	18 (174)

Findings from the pre-FGD questionnaire showed that in total there were 1490 people living in participants’ households. Overall, 65% of the population within study households had access to an ITN, assuming one ITN protects two people. Access was similar across sites, with slightly higher access among study households in the Central Region (72%) compared to participants from the Northern (63%) and Eastern Regions (65%), respectively.

FGD participants represented a range of use practices. Approximately 32% reported being regular users, 23% reported being seasonal users, 43% reported being occasional users, and only 2% reported being non-users in the pre-FGD survey and FGDs revealed nuances beyond these categories. Seven cases were identified from FGD participants across the three sites (Table 2).

**Table 2:** Background characteristics of cases

Case ID	Region	Age	Sex	Type of case
CS1	Central	33	Female	Consistent user
CS2	Central	42	Male	Non-user
CS3	Eastern	38	Female	Consistent user
CS4	Eastern	54	Male	Non-user
CS5	Northern	37	Male	Consistent user
CS6	Northern	28	Female	Seasonal user
CS7	Northern	63	Male	Non-user

### Barriers to consistent ITN use

**Fig. 2:**
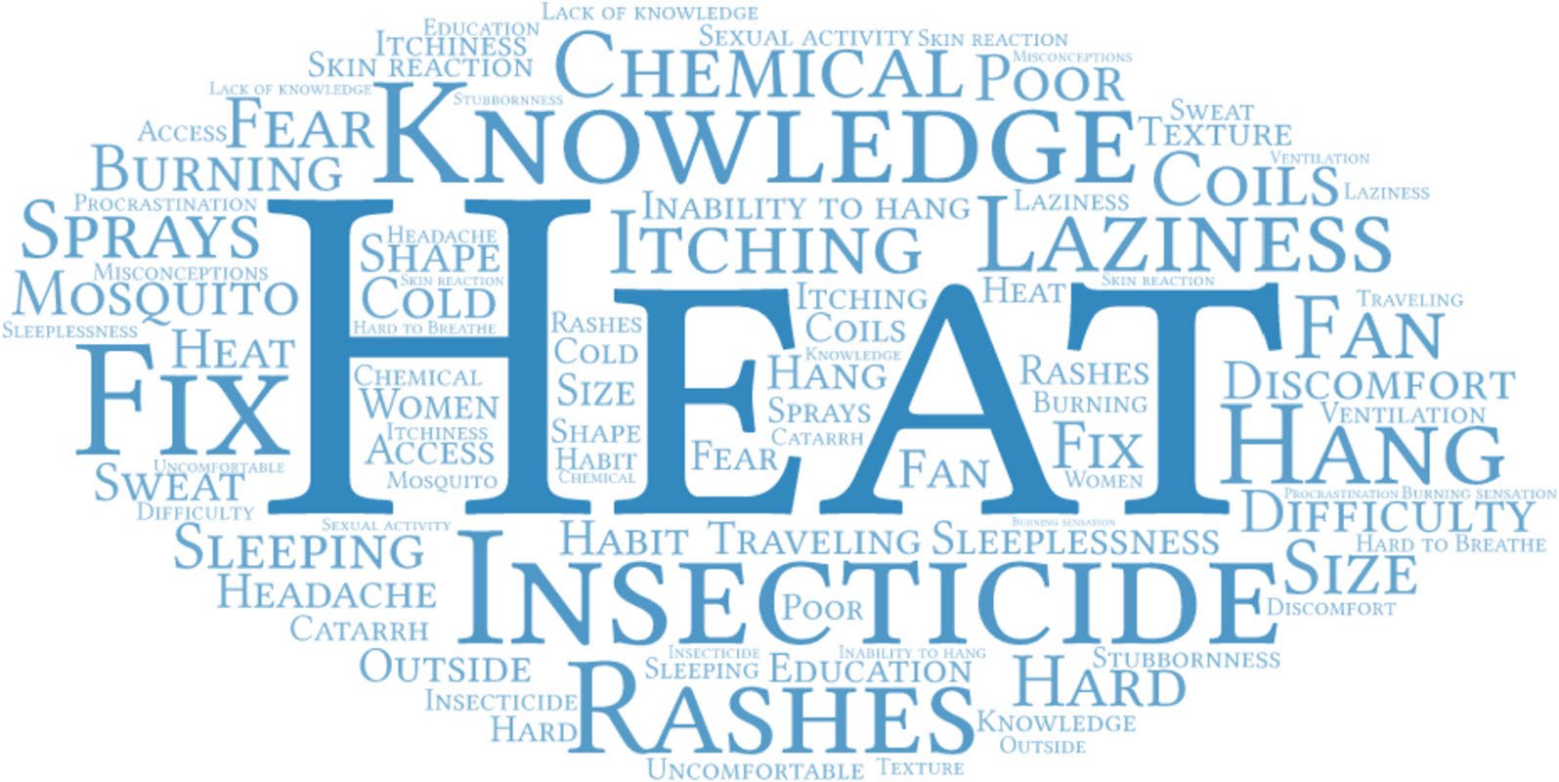
Visual representation (word cloud) of most common barriers to consistent ITN use

Heat and discomfort under the ITN were ranked as top barriers to consistent use across sites, while lack of malaria knowledge and lack of motivation to hang and use the ITN were reported from the Central and Northern Regions. The top ranked barriers to ITN use across sites and FGD categories are included in Table 3 and Fig. 2. Cases represented a spectrum of ITN use profiles –consistent user, seasonal user, and non-user.

**Table 3:** Top ranked reasons for inconsistent use of ITNs from free listing and ranking activity

	Central region	Eastern region	Northern region
Community leaders	Heat Rashes Lack of education	Heat Chemical is too strong and causes reaction The net is short	Heat Reaction to the insecticide Itching
Under-five caretakers	Heat Do not see the need because of mosquito sprays, coils, fan, etc. Laziness to hang the ITN	Heat Reaction to the insecticide Difficulty in hanging the net	Heat Reaction to the insecticide Lack of education
Participants > 49 years	Heat Discomfort ITN material is too hard	Heat Discomfort Reaction to the insecticide	Lack of education Laziness
Health workers	Burning sensation (upon contact) Difficulty in hanging the ITN Heat Lack of education	Heat Reaction to the insecticide Itching	Misconceptions about ITNs Texture and shape of the ITN No access to the ITN by women and children (husband seized the ITN)
Male 18–49 years	Insecticide is too strong Lack of education Inability to hang the ITN	Heat Insecticide is too strong People just don’t like the ITN	Stubbornness Heat Itching/reaction to insecticide
Female 18–49 years	Heat Disruption of sexual activity Burning sensation	Heat Can’t breathe while under the ITN Skin rashes	Heat Laziness Procrastination

Participants across sites reported a number of physical reactions to the insecticide in the ITNs, even after reportedly airing the ITN as instructed during distribution and in some cases, after washing the net. A community leader from the Central Region explained:

*“The chemical they used in the net is quite strong. When you sleep in it some people, and even me for instance, I experience a burning sensation so out of frustration I will move out of the net.”*

A smaller number of participants discussed challenges associated with the design of the ITN. A male community member from the Northern Region described the challenge of hanging a rectangular net compared to a conical net:

*“The round type (conical) is easy to fix and remove but it is distributed in small quantities (distributed less frequently). The four ropes type (rectangular) is difficult to tie, especially when you are tired and in a hurry to sleep.”*

The inconvenience in getting in and out of the ITN in the night was also discussed as a barrier to use. Some participants indicated that sleeping under the net restricted their movement and made it difficult for them to get in and out of bed in the night and this was captured in the narrative below:

*“Once they fix the net and find entering the net uncomfortable, they will not sleep in it again because it did not serve their purpose…In the night, if they want to go and urinate and the net ties them up and they have to remove it, go out and come back to fix it, they feel like they are in prison and will not sleep under it.”*

(Female community leader, Eastern Region, FGD)

The material used in making ITNs was discussed and it emerged that participants have noticed a difference in the materials used to make the ITNs they received at different time-points. A female community member from Northern Region explained:

*“The reason why we are complaining of heat is because the recent one (ITN) they gave us is made of rubber (likely referring to polyethylene). That one produces a lot of heat, so we do not even tie it for the kids. If you try using it, you will end up roasting yourself*.*”*

Some people who received the ITN described as being made of “hard” or “nylon” material, reported using them to make curtains and window screening to prevent mosquitoes from entering their rooms:

*“The one I had following the distribution, frankly speaking, I didn’t sleep in it because it was hard. So I used it for my windows. I fixed them at the back of my windows and even my trap door (a door made of plywood at the lower half and netting material at the top half, usually fixed with a spring that makes it shut automatically), that’s what I used. I have used it as a net for all my windows so that mosquitoes do not enter my room.”*

(Female community leader, Central Region, FGD).

Room size or crowdedness was not commonly cited as a key barrier to ITN use, although some participants explained that the smaller the room the more difficult it was to hang ITNs, especially hanging more than one rectangular net.

### Facilitators to consistent ITN use

**Fig. 3:**
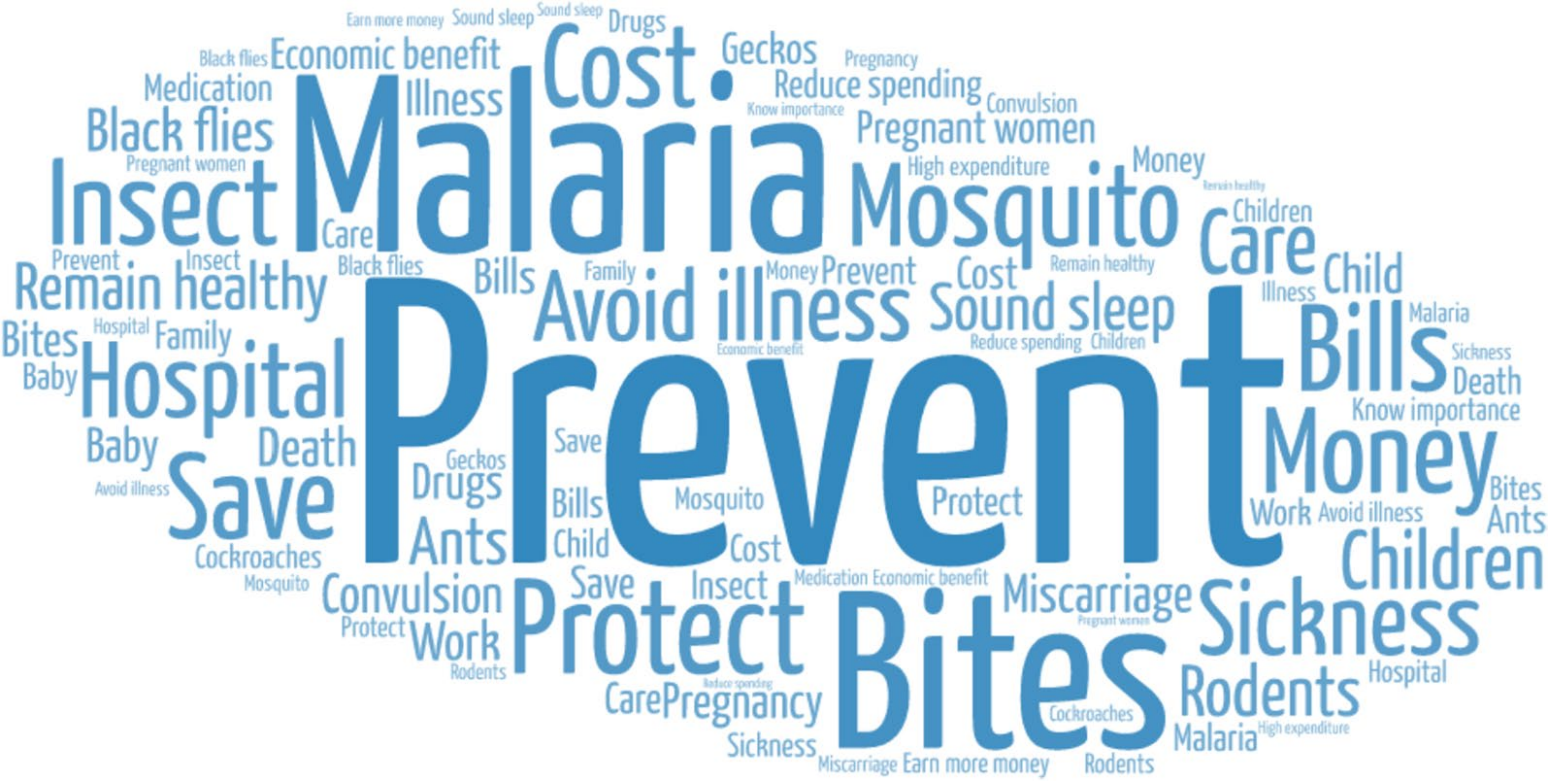
Visual representation (word cloud) of most common motivators to consistent ITN use

Across all study sites, malaria prevention, mosquito bites, saving money, and remaining healthy ranked highest among reasons why people with access to an ITN use it. The top ranked facilitators from the free listing and ranking activity are included in Table 4 and displayed visually in Fig. 3.

**Table 4:** Top ranked facilitators to ITN use from free listing and ranking activity

Type of participants	Central region	Eastern region	Northern region
Community leaders	To prevent malaria To prevent mosquito bites To avoid hospital bills/cost	To prevent malaria Educated about (the purpose of) ITNs To prevent mosquito bites	To protect against malaria To save money from hospital bills and to be healthy to work To protect against other insects, such as ants, flies, and rodents
Caretakers of children under 5 years	To prevent malaria To have a sound sleep To prevent mosquito bites	To prevent malaria To prevent convulsion in children To prevent miscarriage or malaria in pregnancy	To protect against malaria To protect against mosquito bites To remain healthy
Participants > 49 years	To prevent malaria To prevent mosquito bite To have a sound sleep	To protect ourselves To prevent malaria To prevent sickness and death	To prevent malaria To protect against mosquito bites To protect against other illnesses
Health workers	To be healthy To avoid economic cost of health care To prevent mosquito bites	To prevent malaria To protect from mosquito bites To prevent rodents entering the room	To prevent mosquito bites To protect against other insects To save family money
Male 18–49 years	To prevent malaria To prevent mosquito bites To avoid spending money at the hospital	To prevent malaria To prevent insect bites To prevent sickness	To prevent malaria To prevent high expenditure at the hospital To prevent mosquito bites
Female 18–49 years	To prevent mosquito bites To prevent malaria To stay healthy	To prevent malaria To prevent black flies To prevent other sickness	To prevent malaria To prevent mosquito bites To protect against other insects, wild geckos, and cockroaches

While many participants described malaria prevention as a motivating factor, serious first-hand experiences had the most profound impact on ITN use. A female caretaker of a child under five shared how she became a regular ITN user:

*“…I had a baby girl, but we were not sleeping in the mosquito net and she had malaria. She looked very pale…so, we rushed her to the clinic… When we arrived there, she was dead, my baby girl was dead. Since that time, we have slept in the mosquito net every day. Even when it is hot, we sleep in it.”*

(Female caretaker of a child under five, Central Region, FGD)

Some participants drew on their personal experiences, or the experiences of their family and friends, as motivators for consistent use of an ITN. In a case study interview, a man from the Northern Region revealed how he almost died from malaria after he refused to use an ITN. Following that experience, he became a regular user. He explained:

*“I had in the past refused to use the net because of the heat, itching, and other complaints I had heard about. However, I was attacked by malaria and had to be hospitalized for a week and almost died. After being discharged from the hospital, I decided to use the net regularly.*”

While preventing serious illness and death from malaria was of paramount concern, the positive financial impact of preventing malaria was also frequently mentioned. Participants noted that ITN use was a way to save money and ensure they were able to work. A female community member from Eastern Region explained:

*“I have come to understand that if I do not allow my child to sleep in the net he will get malaria. When he gets malaria I too will not sleep and our finances will also come down.”*

Beyond malaria prevention and saving money, a good night’s sleep and preventing nuisance biting was frequently cited across the study sites. Many participants noted that the buzzing sound coupled with bites from mosquitoes and other insects made it difficult for them to sleep soundly. A male community member from Northern Region explained:

*“My motivation for using the net is to prevent mosquito bites and malaria. I also use it to protect myself from the noise the mosquito makes, anytime I hear that noise I am not able to sleep again. So, the net gives me sound sleep.”*

Another female participant from Central Region echoed this sentiment during a case study interview, saying:

“*Oh, the mosquito net, it’s not only mosquitoes that it drives away from the room but also spiders, cockroaches, and other small insects in the room. When any insect gets into contact with it, then it dies.”*

### Seasonality

Participants frequently reported higher ITN use during the rainy season compared to the dry season due to higher perceived risk of malaria, higher density of mosquitoes, and the relative comfort that sleeping under an ITN in cool weather provides. A female community member from Eastern Region described:

“*When the rainy season comes and you sleep in the net, it is really comfortable, and the air too circulates, so when the heat starts to come, then we reject it because…when you sleep in it you will sweat.*”

Participants reported that different factors influence malaria risk across seasons. Participants noted that the risk of malaria is higher during the rainy season; however changes in sleeping patterns, including higher frequency of outdoor sleeping and lower levels of ITN use during the dry season contribute to ongoing transmission in the dry season. A caretaker of a child under five from Northern Region explained the risk during the two seasons this way:

*“Truly malaria affects us twice in a year. During the rainy season, when we have more mosquitoes, and during the time the place is hot because many people will sleep outside because of the heat, exposing them to mosquito bites”*

Temperature differences between urban and rural dwellings were highlighted by a male community member in the Eastern Region. He explained:

*“The heat we are talking about when you are in town, maybe your room has an air conditioner and fan. But, when you come to the villages it is not so, we don’t all have money. So, for the room itself, the sun always shines on the roofing sheets, which makes the room hot in the night. When you enter into the room, it is like you are in a coffin. So, you come out to sleep outside for fresh air until it becomes cool before you go to the room to sleep.”*

(Male community member, Eastern Region, FGD)

In the Northern Region of Ghana, participants revealed that they sleep outside because of the heat and the perception that sleeping outdoors prevents cerebrospinal meningitis (CSM). Hence, sleeping outside is very common during the dry season as described by this community member:

*“Sometime back, when the heat was like this, they say that the heat is too much and that nobody should sleep in the room, because the heat gives us another condition called CSM. That we should sleep (outside) in the compound…and that we should fetch water and drink a lot of water during these hot seasons.”*

(Female community member, FGD, Northern Region)

### Overcoming known barriers to ITN use

#### Outdoor ITN use

Outdoor ITN use was discussed across the three sites. Regular users indicated that they can use an ITN when they are sleeping outside their rooms, in the open-air compound. Participants described a number of local techniques for setting up ITNs outdoors including hanging the net over chairs, filling cans with sand or dirt to hold sticks for hanging the net or hanging the net over a clothes line. For instance, a case study participant from the Eastern Region described her approach to using a net outdoors on her veranda this way:

*“When it is hot inside, we sleep outside here on the veranda* and to prevent mosquito bites we bring the net outside and spread it over *four plastic chairs, when this is done, you can sleep until morning without feeling any heat. So for me, I like to sleep in the mosquito net.”* (CS3)

A limited number of study participants indicated that it is possible for ITNs to be used when a person sleeps outside his/her compound, especially if their work requires that they sleep away from home. Some participants indicated they often use nets when they travel, although this was infrequently reported in this study. A female community leader from the Central Region explained:

*“My fishermen brothers who go for fishing…some of them intentionally decide not to sleep in the net but some of them use their nets when sleeping outdoors.”*

#### Developing a habit of ITN use

Some consistent ITN users noted that once they developed the habit of ITN use, it became easier to continue using it without difficulty. Developing the habit as a child made it easier to consistently use an ITN as an adult. A female case study participant from the Eastern Region described becoming accustomed to net use from a young age, explaining:

*“The reason why I use the mosquito net is that, at first, we were living by the riverside, so when the mosquitoes bit us, we got sick, so they (the government) always brought us the mosquito net. So, when we moved to settle here, I realized sleeping under the mosquito net helps the body as well as the health because no insect will bite you.”*

#### Education on ITN use

Sustained health education emerged as an important factor for increasing ITN use and preventing malaria. The media, television, radio and the use of mobile community information vans and health workers were identified as the main sources of information on malaria in the community. A male case study participant from the Northern Region explained:

*“Continuous education and constant reminders will ultimately bring the desired change we are all clamoring for. Some people are generally difficult and will require more education to change.”*

The importance of images and photos to promote ITN usage was put forward by health workers in the Central Region as a way to ensure people understand the messages that they receive. The potential value of the use of images and photos was explained in the following narrative:

*“I think we should try to put in images to show them… I was presenting and I got an image from the internet, the man had hung the net at the back…of the Toyota pickup truck… and was sleeping … I think when they see the images and all that… it will draw their attention to the fact that they are not the only person with this challenge (of using a net in difficult circumstances) …”*

(Male health worker, Central Region, FGD)

Respondents stated that providing information on how to reduce heat within sleeping spaces could also help increase ITN use. This included addressing sources of heat in sleeping spaces, such as using bulbs that emit low heat, using light curtains, and moving curtains aside during the night to increase access to fresh air as this will reduce the heat associated with sleeping in the net.

### Nighttime activities

Participants perceived gaps in protection before sleeping hours, as well as when away from home. A 61-year-old male community member from the Central Region explained:

*“Sometimes, it is not only about you being in your room. Maybe in the evening by this time around 5:30* *p.m., if you are sitting outside getting some fresh air, even before you go and lie down in the net, the mosquitoes would have bitten you already. Maybe you are at home, eating outside in the evening. Before going to bed the mosquitoes would have bitten you already before you go and use the net. And this happens a lot during the rainy season.”*

(Male community member, Central Region, FGD).

Participants intimated that people of all ages sleep outside of their compounds during funeral rites (wake keeping), religious activities (crusades, conferences), and festivals. For some participants, their work requires that they sleep outside the home. Farming, fishing, and going on night duties were work-related reasons for sleeping away from home. These issues were raised across the study sites. A female community member from Central Region explained:

*“Some of us go for funerals and have to do wake keeping, so we sleep outside the home; we are also engaged in fishing and so we have to go to work, and this will require sleeping outside. It is very common here because it is a fishing community.”*

### Additional malaria prevention methods

Community members employed several strategies to prevent malaria in addition to using ITNs. Participants indicated that they stay indoors in the night and use topical mosquito repellents, coils, aerosol sprays, and fans to protect against mosquito bites and malaria. In addition, at the individual level, people make “trap doors” with nets and use nets as screening material on the windows of their homes to prevent mosquitoes from entering the rooms. At the community-level, participants cited additional strategies used to prevent malaria in the community: clean-up events and clearing bushes around the community to reduce breeding sites for mosquitoes. Participants also identified local remedies to prevent mosquito bites, including the burning of gari (local food made of cassava), ground nut shells, bamboo leaves, orange peels, and other local leaves. The scents and smoke produced from burning these items is perceived to ward off mosquitoes. These local remedies were reported across all the three study sites. A female caretaker of a child under five from the Central Region explained:

*“If we are going to be outside till about 11* *pm, we have to take orange peels, put them in a plate and add some fire and when the smoke disperses, the mosquitoes will not come near and bite us.”*

### Gender and age considerations

Differences were noted across age and sex related to perceived vulnerability, ITN use practices, nighttime activities, and sleeping patterns. Generally, participants stated that children and women go to bed earlier than men. Women and children were often perceived as having a greater risk of malaria and, therefore, more likely to sleep under an ITN. It was generally agreed among participants across the three study sites that men spent more time outdoors at night and were more likely to sleep outside of their compound. A female community member from the Northern Region explained:

*“Men spend a lot of time in the night outside with friends, so they come to sleep late. We, mothers and children, go to bed earlier than them. So, they may be using the net around 12 midnight when they are ready to sleep.”*

A female case study participant, also from the Northern Region, echoed this sentiment explaining:

“*So, in my opinion, a man can freely sleep outside but a woman cannot. She can only sleep in the compound.”*

In the Northern Region, health workers noted a perception in some communities that ITN use was a sign of weakness for men. A female health worker from the Northern Region explained, *“They have this saying, ‘dagban doo bi niŋda’ (meaning a Dagomba man doesn’t do that). So, like they are proud…He feels that he can take care of himself better than using a net because when he uses one, they will say he is just a weakling.”*

Participants noted that the financial burden of sickness is borne mostly by men in the household, while women are the ones that send the sick child to hospitals and stay with them when they are hospitalized. Participants felt that women bear the highest burden of the child’s ill health due to this caretaking role, which serves as a motivator for mothers to promote ITN use in the household. A caretaker of a child under five explained:

*“When the children are sick, we (women) have to carry them to the hospital and the man provides the money. However, if you go to the hospital and the child is admitted, you the mother will have to stay in the hospital and take care of the child. So, for me, I use my net with the children even though their father does not care about the use of the net because when the children are sick, I suffer more.”*

(Female caretaker of a child under give, Northern Region, FGD)

## Discussion

ITNs arguably remain the most effective tool available to prevent malaria; ensuring high levels of access to and use of ITNs is crucial to their success. Among the more than two dozen U.S. President’s Malaria Initiative focus countries that completed DHS or MIS surveys in recent years, Ghana ranks close to the bottom in levels of use among those with access [19]. Understanding barriers and facilitators to use among those with access is critical for closing this gap.

Heat continues to be the most widely reported barrier associated with sleeping under an ITN and a dominant reason why people with access to an ITN choose not to use it at all, occasionally, or seasonally. This finding is consistent with a review of published literature which found discomfort, primarily associated with heat, was a primary barrier to ITN use [24]. Improved housing design that increases airflow may help to reduce heat in bedrooms; additional research on this topic is warranted. Seasonal patterns of ITN use were documented in northern Ghana as early as the 1990s [25, 26]. The seasonal use of ITNs is linked to perceived low mosquito density in the dry season. This perception is supported by entomological monitoring in Ghana which shows higher entomological inoculation rates during the rainy season [27].

Adverse reactions to insecticide were frequently mentioned as inhibitors to ITN use. Changing messaging provided through mass and continuous distribution channels to encourage increased airing time (48 h or longer) prior to first use has the potential to improve user experiences and promote consistent use.

The perceived effectiveness of ITNs in preventing malaria was commonly reported, however, some participants felt nets were more useful in preventing nuisance from mosquitoes, and other pests, for a good night’s sleep. This was also reported in earlier studies where the practical function of mosquito nets may differ from the intended function in some instances [24, 25, 28–31]. Examples of using ITNs for purposes other than sleeping under them were reported in a few instances. When the material of the ITN distributed was perceived as hard, the ITN was more likely to be used for other purposes. Examples of alternative use of ITNs has been documented elsewhere in the literature [32, 33]. A consensus statement put forward by the RBM Partnership to End Malaria classifies alternative use of ITNs in three categories including misuse, neutral repurposing, and beneficial repurposing [34]. Use of a *new* ITN for any alternative purpose is considered misuse, as is use of *any* ITN for fishing. However, use of an inactive ITN for purposes such as covering windows, which can provide some protection from malaria mosquitoes, is classified as beneficial repurposing. National Malaria Control Programmes should consider these practices in development of social and behaviour change messaging.

An important issue to address in ITN promotion is how to encourage ITN use among individuals who spend part or all of the night sleeping outdoors; this was one reason for ‘partial’ non-use reported in our study, and has been reported in earlier studies [24, 28, 35, 36]. Having additional mosquito nets in the household that could be hung in the various sleeping areas, indoors and outdoors, may be helpful. Pulford et al, [24] argued that hanging mosquito nets outdoors may continue to be problematic given current mosquito net designs, which rely on external supporting structures, and proposed the development of ‘outdoor’ or ‘standalone’ mosquito nets that require no external supports yet remain portable and user friendly. Despite the challenge, a number of participants identified local solutions for using their current ITNs outdoors and ensuring a good night’s sleep even during the dry season. Identifying and disseminating local solutions of outdoor ITN use should be considered. Providing visual representations of how to use an ITN outdoors, or in other challenging contexts, should also be explored.

ITN use must be promoted within the larger context of the malaria control and elimination agenda, bearing in mind that no single control tool will be enough to eliminate malaria. While ITNs are an essential tool for malaria prevention, they are designed to primarily protect people while sleeping. A number of studies across sub-Saharan Africa, including in Ghana, have identified activities that occur when malaria vectors are active but net use is not feasible [37–39]. A review of the published literature identified common nighttime activity categories across different contexts in sub-Saharan Africa, including routine social and community events, as well as large scale social events and livelihood activities that can last throughout the night [40]. To this end, personal protection measures, when proven effective, should be promoted among those who have to stay outdoors at night to work or perform other duties [41–43].

There are a number of limitations associated with this study. First, qualitative methods were selected to complement existing quantitative findings and provide a broader understanding of participants’ experiences. The sample size was thus driven by the goal of theoretical saturation, not statistical significance, and must be interpreted accordingly. Further, while self-reported data was utilized to better understand participants’ perspectives, there is the potential for social desirability bias, in which participants report what they perceive as the socially acceptable answer rather than their true behaviour [44, 45]. However, a majority of participants reported that they were not consistent users, and spoke freely about the barriers to using ITNs, suggesting that social desirability bias did not influence the quality of study findings in a meaningful way.

## Conclusions

ITN use, as described by study participants, was not binary (user versus nonuser); one’s ITN use could vary throughout the night, across seasons, and over time. The study results suggest that the greatest gains in ITN use among those with access could be made by promoting consistent use throughout the year among occasional and seasonal users. Opportunities for improving communication messages as well as structural approaches to enhance the usability of ITNs in challenging contexts were identified from this work. Examples include positioning ITN use within the broader context of malaria prevention; increasing the salience of malaria risk; updating messaging to increase airing time before first using the ITN; highlighting the cost and time benefits of prevention over treatment as well as the benefits of a nuisance-free sleep; increasing knowledge of malaria transmission; and identifying and promoting solutions for using ITNs in outdoor or challenging environments. The gender dimensions of ITN use suggest the need to focus on promoting ITN use for all family members and identifying messages and channels that will resonate with both males and females. The results of this study can help to inform social and behaviour change messaging and innovative approaches to addressing the ITN use gap in Ghana.

## Data Availability

The datasets used and/or analysed during the current study are available from the corresponding author on reasonable request.

## Abbreviations

CSM: cerebrospinal meningitis
DHS: demographic and health survey
FGD: focus group discussion
GHS: Ghana Health Service
ITN: insecticide-treated net
MIS: malaria indicator survey
